# Feasibility Study on the Application of Microbial Agent Modified Water-Jet Loom Sludge for the Restoration of Degraded Soil in Mining Areas

**DOI:** 10.3390/ijerph18136797

**Published:** 2021-06-24

**Authors:** Chuning Ji, Jiu Huang, Yu Tian, Ying Liu, Joshua Bosco Barvor, Xintong Shao, Zi’ao Li

**Affiliations:** 1 Engineering Research Center of Ministry of Education for Mine Ecological Restoration, China University of Mining and Technology, Xuzhou 221116, China; jichuning@cumt.edu.cn (C.J.); tiany@cumt.edu.cn (Y.T.); jiasuo199307@gmail.com (J.B.B.); shaoxintong@cumt.edu.cn (X.S.); liziao@cumt.edu.cn (Z.L.); 2 School of Environment and Spatial Informatics, China University of Mining and Technology, Xuzhou 221116, China; liuying340825@163.com; 3 School of Earth and Environment, Anhui University of Science and Technology, Huainan 232001, China

**Keywords:** soil restoration, water-jet loom sludge, microbial agent, prairie mining area

## Abstract

Open-pit mining causes soil damage and affects the health of the ecosystem. In the arid grassland mining areas, the soil is severely sanded, water-starved, and saline, thus making it difficult for plants and microorganisms to survive. Water-jet loom sludge can be used to improve the quality as it contains a lot of clay and is rich in organic matter, which provides a material basis for microorganism activity. To explore the effects of microbial agent-modified water-jet loom sludge on the restoration of degraded soil in grassland mining areas, four pot trials were set up, i.e., for untreated soil, the application of a microbial agent alone, the application of water-jet loom sludge alone, and the combined application of water-jet loom sludge and the microbial agent. The results show that (1) microbial agent-modified sludge can improve soil water-holding capacity and aggregate stability; (2) the nutrient content of the restored soil fraction increased significantly, and the pH of the original saline soil decreased from 9.06 to 7.84; (3) this method significantly increased plant biomass and microbial biomass carbon and enhanced the abundance and diversity of fungi and bacteria. The three treatments had different results in different soil properties, and the effect of the combined water-jet loom sludge and microbial agent treatment on soil remediation was significantly better than the individual application of either.

## 1. Introduction

The development of coal industry has made a great contribution to the economic development of China [1], but coal mining also leads to direct damage to environmental health, such as the loss, collapse, and occupation of land, as well as indirect damage via the disorder of the mining area ecosystem [2]. For the prominent soil degradation caused by high-intensity and large-scale coal mining coupled with the reality that the ecological restoration rate of China’s coal mining areas is less than 1/4, achievement of effective and low-cost soil restoration in mining areas has become a very urgent task in China [3]. At present, the country implements strategies such as level-by-level control and zoning restoration, emphasizing that natural restoration and artificial restoration complement each other [4]. In 2020, the National Development and Reform Commission (NDRC) and the Ministry of Natural Resources (MNR) of China issued the “Master Plan of Major Projects for the Protection and Restoration of Important National Ecosystems (2021–2035),” emphasizing the restoration of mine ecology in the key ecological zone and the implementation of integrated management of terrain reshaping, soil reconstruction, and vegetation rebuilding [5]. In recent years, for the reclamation of open-pit mines, integrated mine ecology restoration, soil reconstruction, geomorphological reshaping, and rapid revegetation methods have been proposed and are widely used in large mining areas [4,6,7]. However, although the country has been promoting soil restoration in open-pit mines in arid and semi-arid areas, there are still such phenomena as “high cost” and “reliance on artificial irrigation” [8]. The cause of formation is that under dry climate conditions, the topsoil used for mulching features a large particle size and a lack of nutrients, so there is no suitable soil environment for microorganisms to survive, which in turn leads to non-decomposition of litter, discontinuous ecological processes, low soil function, and difficulty in vegetation survival and propagation [9,10]. Therefore, a topsoil remediation material that can neutralize salinity and improve soil quality is urgently needed to provide a place and nutrients for microbial activities and build a good soil environment for the normal development of native plants.

The annual discharge of water-jet loom wastewater in China is about 2.37 × 10^9^ t. Water-jet loom sludge is generated during textile wastewater treatment [11]. Unlike traditional shuttle weaving methods, a water-jet loom uses a membrane bioreactor process involving a high-pressure water jet to spin the weft. It also provides a new wastewater treatment process that combines membrane separation technology and biotechnology, and the remaining solid waste forms the water-jet loom sludge. Being a high-emission solid waste, landfills, incineration, and sea dumping treatment methods are limited by its load and high cost [12]. Therefore, increasing attention has been given to the application of sludge in agriculture and land reclamation [13]. In Britain, France, Switzerland, Sweden, and the Netherlands, the agricultural usage rate of urban sludge is as high as 40–50% [14,15]. In China, the policy stipulates subsidies for sludge treatment or disposal; for example, in Suzhou, the local comprehensive sludge disposal subsidies are 61–77 $/t. Water-jet loom sludge contains a collection of microbial micelles formed by a variety of microorganisms and their adsorbed organic and inorganic substances [16]. It can form a large amount of humus [17], change the existing form of heavy metals, and improve the soil organic matter, structural characteristics, and water retention capacity of the soil through the action of microbial colonies. Thus, it is expected to be a good soil improver, a viscous mineral-rich raw material, and an organic fertilizer, and can be used in sandy soil reclamation in grassland mining areas.

Many researchers worldwide have studied the sludge improvement of degraded soil [18,19,20,21]. Ciolea studied the possibility of turning sterile soil into a fruitful one by using sludge with different physical and chemical properties [22]. Paula used wastes from the pulp and paper industry which were co-granulated to improve the quality of acid mine-contaminated soils with high Cu, Pb, and Zn concentrations [23]. Fernandes [24], Zerzghi [25], Criquet [26], and others have also studied the improvement of soil quality, microbial activity, and enzyme activity by applying sludge. However, the dump formed by open-pit mining in arid grassland area was covered by stripped topsoil. The destruction of the soil structure as well as vegetation seriously affects the species quantity and activity of microorganisms [27,28]; sludge alone cannot achieve the remediation requirements in seriously degraded soil [29].

Research on soil microorganisms in mining areas mainly focuses on the change in the community structure and the quantitative characteristics of microorganism, and some scholars put forward the method of using microorganisms to repair mines [30]. For example, Zhang et al. found that arbuscular mycorrhizal fungi (AMF) inoculation may play an active role in promoting plant growth and improving soil quality in the long term and is conducive to the rapid ecological restoration of damaged mining areas [31]. However, the application of microorganisms alone cannot change the physical structure and texture of soil in a short period of time; moreover, it is difficult to improve soil water-holding capacity in arid areas. Therefore, according to the soil degradation characteristics of Inner Mongolian steppe mining areas, a pot trial was designed to explore a combination of a microbial agent and water-jet loom sludge for more effective soil remediation in this semi-arid steppe area.

## 2. Materials and Methods

### 2.1. Study Region and Sampling

Soil samples were collected from the Shengli open-pit mining area of the City of Xilingol, Inner Mongolia (43°57′–44°14′ N, 115°30′–116°26′ E). The mining area is divided into 10 minefields, including six open-pit coal mines, one open-pit germanium mine, and three underground coal mines. The average annual precipitation is 336.9 mm and the annual evaporation is 1600–1800 mm, which is 4–5 times the average rainfall. The main zonal soil in this area is chestnut-colored soil with a sandy soil texture, which is prone to sand and wind erosion, and the ecological environment is fragile. The vegetation is mainly composed of bunch grass and rhizomatous grass, including *Stipa grandis*, *Leymus chinensis*, *Agropyron cristatum*, and *Setaria viridis*. The soil was sampled from each plot by collecting 20 randomly selected cores (0–10 cm deep) that were thereafter well-mixed and homogenized, and these samples were placed in sealed bags. The samples were evenly mixed and the stone and plant residues were removed, and then some of them were stored in a 4 °C refrigerator for microbial biomass carbon (MBC) estimations. After natural air drying, the samples were screened through a 100-mm mesh for physical and chemical properties determination (Table S1) and the pot trial.

**Figure 1:**
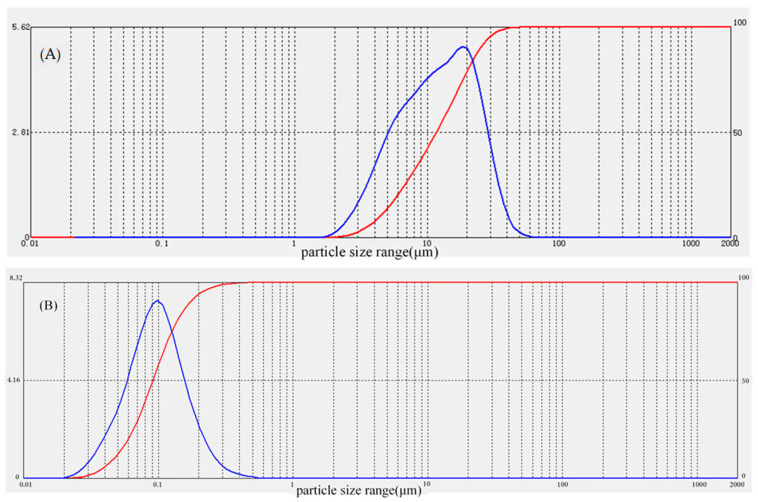
Particle size of the degraded soil (**A**) and water-jet loom sludge (**B**). The red line represents the differential, that is, the proportion of the individual particle sizes; the blue line is the cumulative curve representing the percentage of the previous grain size accumulation. The vertical axis represents the percentage content and the horizontal axis represents the particle size range. The particle size distribution pattern and law can be seen intuitively.

Water-jet loom sludge samples were collected from the riverside of Wujiang District, City of Suzhou. The pH of the samples was measured on the day of collection; some of the samples were air-dried and any impurities were removed. They were then milled with a mortar and sieved through a 100-mm mesh sieve before the available N (AN), available P (AP), available K (AK), total N (TN), total P (TP), mineral K (MK), soil carbon content (SOC), heavy metal content, and particle size were measured (the chemical properties of the sludge are shown in Table S1, particle size of the sludge (B) and degraded soil (A) are shown in Figure 1). The microbes selected in this experiment were composed of *Bacillus* spp., yeast, and photosynthetic bacteria (Beihai Qiangxing Biotechnology Co., Ltd., Guangxi, China), with the effective viable count of CFU > 0.5 billion/g.

### 2.2. Pot Trial and Harvest

According to the USDA classification system of soil texture (Figure S1) [32], the texture of the soil samples and water-jet loom sludge (mixed at a ratio of 2.5:1) was determined to be loam by a hydrometer. The pot trial was conducted in April 2019 in the laboratory of the School of Environment and Spatial Informatics, China University of Mining and Technology. In the pot trial, four different groups of treatments were set up (Table 1). Five parallel experiments were set up for each of the four treatments, and all the parallels were randomly arranged for a total of 20 experimental groups. We mixed the soil and sludge thoroughly using a mortar, and then put the samples into plastic pots with drainage holes (top diameter, 30 cm; bottom diameter, 40 cm; height, 35 cm; with a hole at the bottom and a pot holder at the bottom to prevent soil leakage); each pot was filled with 2.5 kg sample, and the substrate volume was 90% of the total volume of the pot. Two groups of 10 pots of S + M and S + SL × M were mixed with equal amounts of mixed microbial agents (3 g powder was added to 1000 mL distilled water and shaken for 0.5 h to activate, and 10 equal parts were added to the distilled water sprinkled on the same day). After 10 d of soil stabilization, *Setaria viridis* seeds of uniform size and full grain were selected, disinfected with 10% H_2_O_2_ for 5 min, and rinsed five times with deionized water, and the seeds were soaked using deionized water for 24 h. Each pot was sown with 10 germinated seeds that had been soaked, and after 7 d of incubation (growing about 15-cm seedlings), the pots were moved to an open area outdoors, and each pot was taken under consideration. The pots were managed consistently and harvested on 4 August. During this period, distilled water was added by sprinkler irrigation to maintain the water content of 60% of the field water-holding capacity, depending on the actual conditions and weather. CH was measured every two days from 5 May to 31 July. In early August, CH of *Setaria viridis* did not vary by more than 1 cm for eight consecutive days. After harvest on August 4, whole plants and inter-root soil were collected. The wheat seedling plants were washed with tap water and deionized water repeatedly, three, four, or five times, and the plants were divided into stems, leaves, and roots, dried, and put into an oven to kill at 105 °C for 30 min and then baked at 65 °C until constant weight. The DNA sequences of soil microorganisms, soil water-holding properties, agglomerate properties, and chemical properties were determined after harvesting.

### 2.3. Experimental Methods

The Illumina Miseq platform was used to amplify and sequence the fungal and bacterial DNA of each treatment soil in the experiment. Based on the barcode sequences and PCR amplification primer sequences, the raw sequences were spliced and filtered using the QIIME software to obtain valid data. The valid data of all samples were analyzed by USEARCH according to the standard procedure and clustered into operational taxonomic units (OTU) with 97% sequence similarity. Chao1 and Shannon’s diversity index were calculated for each sample to compare the differences in soil bacterial and fungal α-diversity between treatments [33].

The mean weight diameter (MWD) [34] and the geometric mean diameter (GMD) of soil agglomerates [35] are calculated as follows:

$$MWD=\frac{\sum_{i=1}^{n}{\bar{x}}_{i}w_{i}}{\sum_{i=1}^{n}w_{i}}$$

$$GMD=exp\left[\frac{\sum_{i=1}^{n}w_{i}\ln{\bar{x}}_{i}}{\sum_{i=1}^{n}w_{i}}\right]$$

where $w_{i}$ is the ratio of the weight of agglomerates at particle size *i* to the dry weight of the soil sample; ${\bar{x}}_{i}$ is the average diameter of agglomerates at particle size *i*.

The soil agglomerate fractal dimension (D) is calculated as follows [36]:

$$\frac{M(r<{\bar{x}}_{i})}{M_{T}}={\left(\frac{{\bar{x}}_{i}}{x_{max}}\right)}^{3-D}$$

The logarithm of both sides of the equation:

$$\mathrm{lg}\left[\frac{M(r<{\bar{x}}_{i})}{M_{T}}\right]=\left(3-D\right)\mathrm{lg}\;\left(\frac{\bar{X_{i}}}{X_{max}}\right)$$

where $\bar{X_{i}}$ is the average diameter of particle size *i* agglomerates; $M\;(r<{\bar{x}}_{i})$ is the weight of the agglomerates with particle size less than $\bar{X_{i}}$; *M_T_* is the total weight of the agglomerates; *X_max_* is the maximum particle size of the agglomerates.

Other assays involved in the experiments are shown in Table S2.

### 2.4. Data and Statistical Analysis

Analysis and visualization of high-throughput sequencing data were done in the R (version 3.6.0) environment. The alpha diversity index of the samples was calculated using the QIIME software (version 1.9.1). Distance-based redundancy analysis (dbRDA) of biological properties and soil chemistry was performed using the vegan package (version 2.2.5) [37]. Statistical analysis was performed using SPSS to calculate the minimum, the maximum, the mean, and the standard deviation for all indicators, and Kolmogorov–Smirnov test was used to determine the normality of the data [38]. All the indicators met normal distribution except MK, which was single-peaked and log-normal. To satisfy the assumption of normality for the ground statistical analysis, MK was log-transformed and transformed back in SPSS. The LSD method (*p* < 0.05) was used for the significance of differences test, and Origin was used for the graphs.

$$LSD=t_{p/2}\sqrt{MSE\left(\frac{1}{n_{i}}+\frac{1}{n_{j}}\right)}$$

where *t_p_*_/*2*_ is the critical value of *t*-distribution, which is obtained by checking the *t*-distribution table with degrees of freedom of *n*–*k* where *n* is the total number of samples and *k* is the number of different levels in the factors; *MSE* is the intraclass variance; *n_i_* and *n_j_* are the sample sizes of the *i*^th^ and *j*^th^ samples, respectively [39].

## 3. Results

### 3.1. Effects of Sludge and/or Microbial Agents on Physical Properties of the Soil

**Figure 2:**
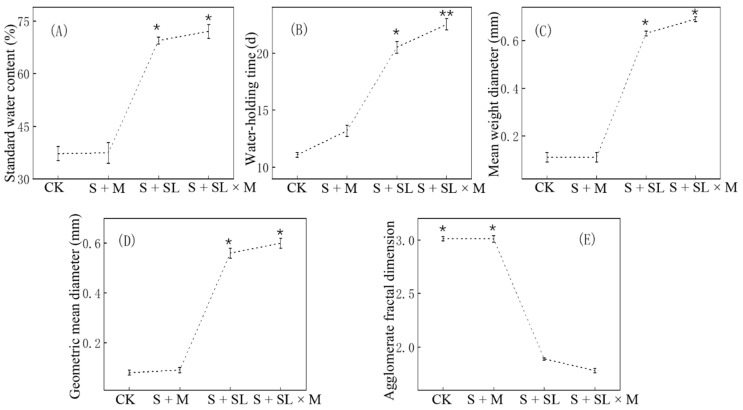
Changes in physical properties of the soil after the addition of water-jet loom sludge and/or a microbial agent; (**A**) saturated water content of differently treated soil; (**B**) water-holding time of differently treated soil, thus reflecting the soil’s water-holding capacity; (**C**) mean weight diameter of differently treated soil; (**D**) geometric mean diameter of differently treated soil; (**E**) soil agglomerate fractal dimension. CK refers to the untreated mining soil samples, S + M refers to the mining soil samples with the microbial agent, S + SL refers to the mining soil samples with water-jet loom sludge samples mixed at a ratio of 2.5:1, and S + SL × M refers to the mining soil samples with water-jet loom sludge samples and the microbial agent. Asterisks indicate statistically significant differences (* represents *p* < 0.05. ** represents *p* < 0.01).

The combination of sludge and a microbial agent changed the physical properties of the soil significantly. As shown in Figure 2, the trends in the saturated water content, water-holding time, MWD, and GMD under the four treatments were similar, and the trend of the soil agglomerate fractal dimension (D) was just the opposite. In the rainfall infiltration simulation experiment, the saturated water content of the original soil sample (CK) was 37.2%, while the water-holding time was 11.1 d. The saturated water content of the experimental treatments containing sludge was 1.85 times that of CK, and their water-holding times were 1.84 times that of CK. Among them, S + SL × M had the highest values (*p* < 0.01), 4% and 10% higher than those of S + SL. This shows that the application of sludge significantly improves the water-holding capacity of the soil, and the application of microorganisms also has an impact on the physical structure of the soil. The MWDs and GMDs of S + SL and S + SL × M were significantly higher than those of CK (*p* < 0.05), and the S + SL × M value was the highest. The MWD of the S + M soil with only the microbial agent was 0.11 mm. The results show that the application of the water-jet loom sludge increases the stability of soil aggregates. The soil agglomerate D value decreased significantly after sludge application, with the lowest value of 1.78 in S + SL × M, indicating that water-jet loom sludge application can increase soil agglomerate stability and soil agglomerates shift from small to large particle size.

In 2012, Inner Mongolia issued a local standard to determine the grassland soil moisture level index (Table S3) [40], and the saturated water content of the soil was used to express the relative humidity of the soil [41]. The soil treated by water-jet loom sludge can reach the second class of soil moisture, and the soil repaired by the microbial agent and sludge can reach the first class of soil moisture. These two types of soil have the characteristics of loam soil. The application of a microbial agent alone cannot improve the relative humidity of the soil, and just like CK, it can only reach the third class of soil moisture.

### 3.2. Effects of Sludge and/or a Microbial Agent on Chemical Properties of the Soil

As shown in Table 2, pH of the soil after the application of water-jet loom sludge decreased from 9.06 to 7.91, and pH of the soil with only the microbial agent increased slightly. The SOC content was higher after the application of the microbial agent alone (compared to CK), and it was 56% higher than CK in case of the S + SL × M treatment. The results show that both the S + M and S + SL treatments increased the SOC content, but the effect of the combined treatment was much better. The SOC, TN, TP, and AK contents of the S + SL soil were significantly higher than those of CK (*p* < 0.05), while the S + SL × M treatment significantly increased the abovementioned indexes. The soil’s AN and AP were more sensitive to water-jet loom sludge application. They were 12.0 and 18.33 times higher in the S + SL treatment and 12.2 and 22.14 times higher in the S + SL × M treatment than in CK. However, the improvement of soil fertility by the S + M treatment was relatively weak: only the SOC increased significantly compared to CK. Both TN and SOC were significantly higher in S + SL × M and S + SL than in CK and S + M, but the C/N ratio was lower due to a greater proportion of elevated TN. The soil’s C/N ratio was significantly different between the groups with and without sludge application. This situation is similar to 3.1 where the addition or not of sludge also caused significant differences in physical properties of the soil so that changes in soil aggregates and porosity may have caused the soil’s microbial activity to be enhanced, thus promoting N mineralization [42,43]. It is worth further exploring.

### 3.3. Effects of Sludge and/or Microbes on Biological Characteristics of the Soil

**Figure 3:**
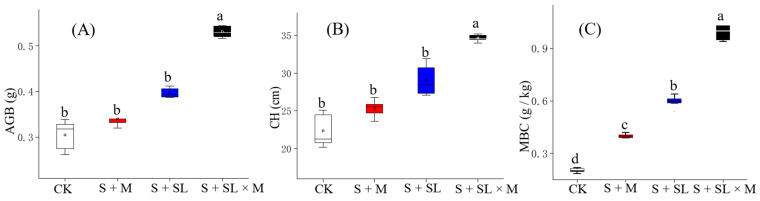
AGB (aboveground biomass) (**A**), CH (canopy height) (**B**), and MBC (microbial biomass C) (**C**) in the soil after harvesting at the same planting time for different treatments; different letters show significant differences between the mean values (*p* < 0.05). CK refers to the untreated mining soil samples, S + M refers to the mining soil samples with the microbial agent, S + SL refers to the mining soil samples with water-jet loom sludge samples mixed at a ratio of 2.5:1, and S + SL × M refers to the mining soil samples with water-jet loom sludge samples and the microbial agent.

The canopy height (CH) of each plant sample was measured, and the average CH in each pot was calculated and recorded. Meanwhile, the AGB was calculated according to the dry weight of the plant roots and leaves. CH and AGB of *Setaria* *viridis* in the pot trial are shown in Figure 3A,B. AGB, CH, and MBC showed the same trend, and the S + SL × M treatment significantly enhanced all three indicators (*p* < 0.05). The combined application of the microbial agent and water-jet loom sludge was able to enhance plant biomass 1.7-fold, plant height—1.6-fold. Furthermore, MBC was significantly different in all the four groups of treatments (Figure 3C), indicating that microbial activity was sensitive to changes in all the three treatments and was the highest in S + SL × M. Single application of the microbial agent or sludge elevated CH and AGB, but not significantly.

Chao1 was used to characterize the abundance of soil bacteria and fungi, while Shannon’s index was used to characterize the diversity of soil bacteria and fungi (Table 3). There were significant differences in Chao1 of bacteria between CK and S + M and S + SL × M, and the bacterial abundance was enhanced by 134.8% and 347.1% respectively, compared to CK. S + SL was able to enhance Chao1 compared to CK, but the differences were not significant. The Chao1 index of fungi was significantly different in all the four treatment groups, indicating that both sludge and fungicide applications increased the abundance of fungi. Compared to CK, S + M increased the diversity of bacteria and fungi, while the S + SL treatment had almost no effect on the diversity of bacteria and fungi. However, compared to S + M, S + SL × M resulted in a more significant enhancement of the diversity of soil bacteria and fungi. This indicates that single application of sludge did not enhance microbial diversity in degraded soil, but combined application of sludge and microorganisms enhanced microbial diversity more significantly, which may have been due to the fact that organic matter in sludge provides nutrients for the survival and expansion of some microorganisms or may have also been related to the change in soil texture and structure.

### 3.4. Correlations between Biological Characteristics and Chemical Properties of the Soil

The Pearson correlation of the chemical properties and biological properties of the soil in the experiment showed that biochemical properties of the soil were closely related. The soil’s pH was significantly negatively correlated with the concentration of available N, P, and K. It also affected the SOC and TN, which in turn affected the soil’s C/N ratio. The soil’s C/N ratio was significantly negatively correlated with the soil’s TN concentration and was not significantly correlated with SOC. The AGB and CH of *Setaria* *viridis* were significantly positively correlated with each other and were also significantly influenced by TN, TP, and SOC. MBC was negatively correlated with pH, indicating that the originally strongly alkaline soil had a significant inhibitory effect on microbial activity, while MBC was positively correlated with CH and AGB of *Setaria* *viridis*, and AP, AK, TN, and TP also positively influenced soil’s MBC.

There was a significant positive correlation between AGB and CH (*p* < 0.01), and both were affected by the SOC and MBC. In terms of soil nutrients, AGB depended on the TN and TP, while CH was significantly correlated with AK, TN, and TP (*p* < 0.05).

**Figure 4:**
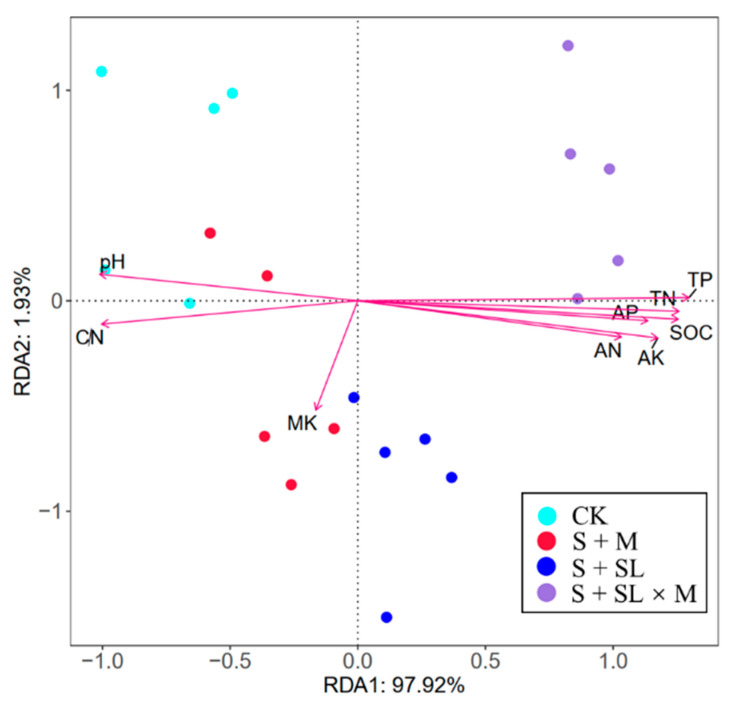
Redundancy analysis (RDA) of biological characteristics and chemical indicators of the soil for different treatments (permutation test: *p* = 0.001).

RDA of biological properties (CH, AGB, MBC) and chemical properties of the soil after different treatments was performed to describe the correlation between differences in biological properties and chemical properties of the soil between the samples (Figure 4) [44]. The results showed that the RDA1 and RDA2 axes explained 97.92% and 1.93% of the results, respectively, i.e., the nine environmental factors cumulatively explained 99.85% of the plant and microbial properties in the first two axes. The presence or absence of sludge application resulted in differences in biological characteristics, with S + SL × M and S + SL distributed on the right side of the RDA1 axis, CK and S + M—on the left side of the RDA1 axis. TP, TN, AK, TP, AN, and AP had the greatest effect on the biological characteristics of S + SL × M and these factors were highly positively correlated with each other, which was consistent with the results of the correlation matrix (Table 4). The changes in biological characteristics were mainly driven by the soil’s nutrient status and pH, with *p*-values less than 0.001 for all soil environment factors except MK, which reached a significant level.

## 4. Discussion

**Figure 5:**
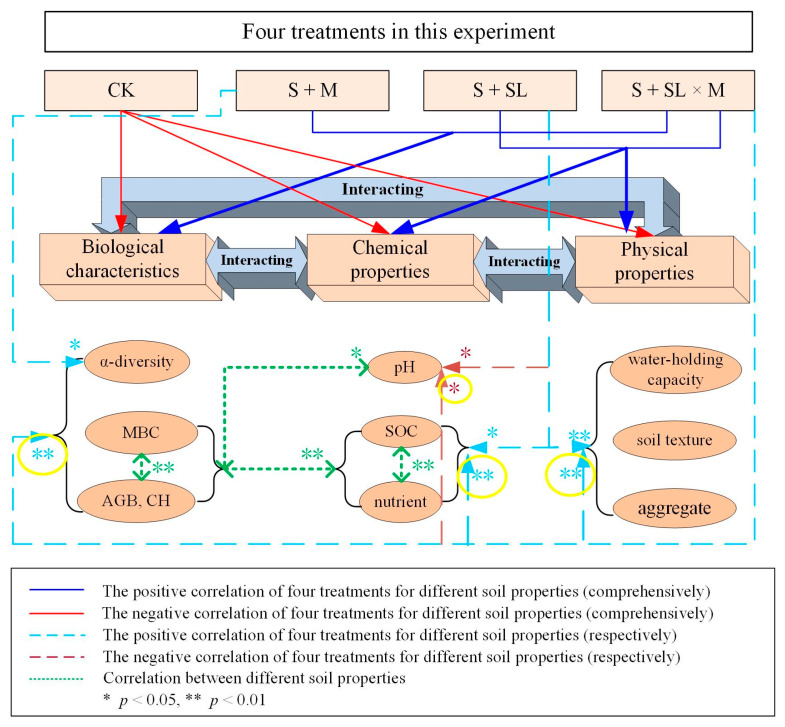
Correlation between different treatments and soil properties.

In recent years, land degradation in China’s grassland mining areas has raised widespread concern. Soil degradation around the Inner Mongolian mining areas has been serious [45]. As Ajayi et al. mentioned, this kind of sandy soil requires viscous mineral-rich raw materials as improvers [46], and, in accordance with the research results of Wu, there are large amounts of silt and clay in water-jet loom sludge [47]. In this study, water-jet loom sludge can therefore improve the organic matter content, the structural characteristics, and the water retention capacity of degraded soil (Figure 5).

The soil repaired by a microbial agent and sludge can reach the first class of soil moisture. In that case, the herbage of meadow steppe and typical steppe turns green and grows well in spring, while that of desert grassland grows well. In summer, forage grass grows vigorously, and the coverage of most grasslands reaches 61–80% in summer and autumn. In autumn, all kinds of grassland’s regenerated forage grow normally. It is suitable for grazing and feeding of all kinds of livestock [47]. The soil’s physical property results show that the S + SL and S + SL × M treatments significantly increased the saturated water content and water-holding time of the soil, while the effects of the S + M treatment were not significant. Meanwhile, MWD and GMD of the soil were significantly improved, and the soil agglomerate fractal dimension was significantly reduced after water-jet loom sludge application. In the simulated rainfall infiltration and evaporation experiments in this study, the soil’s water-holding capacity in case of S + SL and S + SL × M was greatly improved, and the saturated water content was at least 1.85 times that of CK. This means that water-jet loom sludge contains a large amount of clay and silt, as well as rich organic matter, so that the soil is able to form agglomerate structures with water retention and fertility, and the stability of soil aggregates increases [48]. The restored soil has the binder needed for the formation of soil agglomerates, which in turn increases the number of agglomerates in the soil, improves soil agglomerate stability, and tightens the soil structure. Menon and Manoj’s research shows that in the soil with an aggregate structure, there are ventilation pores (non-capillary pores) between the aggregates, which can be permeable and ventilated, and a large amount of surface runoff can be rapidly absorbed into the soil [49]. Some studies have shown that few non-capillary pores exist and water permeability is poor in single-grain or large-block structured sandy soil. Slightly more rainfall flows along the surface, causing water and soil loss. However, the internal soil still cannot absorb enough water, and soil drought occurs soon in sunny weather. In this study, for the environment of the arid grassland mining area, using microbial agent-modified sludge, the restored soil structure formed a large number of capillary pores, thus allowing the capillary water to move faster and supply water continuously [50].

Application of sludge could significantly enhance the nutrient content of the soil. However, it is worth noting that MK in the soil of the three treatments was not significantly higher than that of CK. Their content in the normal Inner Mongolian grassland soil is relatively low, so we propose that the method described in this paper is used with a K fertilizer when improving the soil. Moreover, the C/N ratio can reflect the characteristics of microbial mineralization and sequestration of N during the decomposition of organic matter [51]. The soil’s C/N ratio is also regulated by microorganisms, and the remediation of microbial agent-modified sludge resulted in corresponding changes in the number and activity of microorganisms in the soil, which eventually led to a decrease in the soil’s C/N ratio [52]. This may be due to the increase in porosity in the restored soil aggregates, the adequate oxygen supply inside the aggregates and the oxidation and reduction equilibrium of the soil [53], the increased activity of aerobic microorganisms, resulting in the accelerated decomposition of organic C and the increased release of effective N, thus reducing the C/N ratio [54]. The soil’s pH decreased from 8.96 to 7.91 and 7.83, respectively, after applying sludge and microbial agent-modified sludge. This could inhibit soil salinization, hardening, and other unfavorable phenomena in the study area. RDA showed that the soil’s nutrient profile and pH mainly drove the growth status and microbial activity of *Setaria* *viridis*. The soil’s pH affects the soil’s microbial community and enzyme activity. Therefore, the application of sludge plays a neutralizing role in the salinization and alkalinity of degraded soil in a mining area, forming the necessary conditions for the expansion and activity of soil microorganisms and playing a key role in the continuity and stability of the internal ecological processes of the soil [55].

In the experiment, the S + M treatment significantly increased soil’s MBC (*p* < 0.01). This indicates that the application of the microbial agent increased the biomass of soil microorganisms. However, S + SL × M increased soil’s MBC by 15.0%, which could be related to changes in the physical structure of the soil, according to the research conclusions of some scholars [56]. The aggregate structure of the S + SL × M soil is exactly the environment required for the effective reproduction and activity of soil microorganisms. Microbial quantity and activity are affected by the structure, aeration, water status, nutrient status, and other factors in the soil. This was also confirmed by the variation of fungal and bacterial Chao1, Shannon’s index, and MBC in the soil.

Due to the low content of heavy metals in water-jet loom sludge, the heavy metal concentration in the soil was diluted after mixing. Unlike urban sludge, fly ash, and several other raw materials [57], the use of water-jet loom sludge as a raw material for soil remediation effectively avoids secondary pollution.

This study focused on the effects of water-jet loom sludge and a microbial agent on the physical and chemical properties of degraded soil in grassland mining areas, providing a novel idea for the restoration and reclamation of degraded land in grassland mining areas. However, the long-term effects of the sludge combined with a microbial agent on the physical and chemical properties of the soil need further study. In addition, the study of the biological characteristics of the soil can be further improved by including the activity of soil enzymes, the composition and diversity of the microbial community [58], the microbial groups around the root system of the plants after growth, etc. In the future, it will be necessary to carry out a large-scale field test in the research area to examine the applicability of water-jet loom sludge and the microbial agent in the local area as well as the long-term effects of soil improvement. This will allow us to study the restoration of degraded soil in grassland mining areas more accurately and comprehensively.

## 5. Conclusions

Mining has seriously damaged the soil structure of Inner Mongolia grassland; vegetation cannot be naturally restored, which has seriously harmed environmental health. The combination of water-jet loom sludge and a microbial agent can reclaim the damaged soil in terms of the physical, chemical, and biological properties of the soil. Sludge can neutralize the saline–alkali soil, provide nutrients, and enhance aggregate stability and soil water retention. The results showed that the combined application with a microbial agent had the best effect on soil remediation, which could significantly increase soil microbial biomass carbon, bacterial and fungal abundance and diversity, and also enhance the *Setaria* *viridis* height and biomass. This economical remediation method can not only make full use of the high-emission solid waste, but also significantly improve the soil’s performance, which is difficult to achieve with the existing artificial management methods, and is also fundamental to accelerating the restoration of grassland ecosystems.

## Figures and Tables

**Table 1:** Pot trial design.

Treatment	Soil (g)	Water-Jet Loom Sludge (g)	Microbial Agents (g)	Number of *Setaria* *viridis* Seeds	Number of Repeated Trials
CK	2500.00	0.00	0.00	10	5
S + M	2500.00	0.00	0.25	10	5
S + SL	1785.00	715.00	0.00	10	5
S + SL × M	1785.00	715.00	0.25	10	5

**Table 2:** Effects of different treatments on chemical properties of the soil.

Treatment	pH	SOC (g/kg)	TN (mg/kg)	TP (mg/kg)	MK (mg/kg)	AN (mg/kg)	AP (mg/kg)	AK (mg/kg)	C/N Ratio
CK	9.06 ± 0.02 ^a^	3.6 ± 0.01 ^d^	458.0 ± 9.23 ^d^	344.0 ± 22.0 ^d^	2.18 ± 0.01 ^b^	8.72 ± 0.16 ^b^	1.5 ± 0.00 ^d^	33.0 ± 2.00 ^d^	7.90 ± 0.02 ^a^
S + M	9.14 ± 0.00 ^a^	7.25 ± 0.32 ^b^	893.0 ± 13.92 ^c^	666.0 ± 1.55 ^c^	1.99 ± 0.02 ^c^	10.47 ± 0.28 ^b^	2.9 ± 0.02 ^c^	45.6 ± 8.07 ^c^	8.11 ± 0.05 ^a^
S + SL	7.91 ± 0.04 ^b^	15.83 ± 0.19 ^c^	2127.7 ± 33.7 ^b^	998.2 ± 15.2 ^b^	2.98 ± 0.11 ^a^	104.64 ± 16.6 ^a^	27.5 ± 1.9 ^b^	90.6 ± 0.50 ^b^	7.40 ± 0.02 ^b^
S + SL × M	7.84 ± 0.02 ^b^	20.65 ± 3.2 ^a^	2898.9 ± 34.7 ^a^	1509.2 ± 47.6 ^a^	1.77 ± 0.04 ^c^	106.18 ± 12.0 ^a^	33.2 ± 0.05 ^a^	99.6 ± 4.80 ^a^	7.12 ± 0.02 ^b^

Note: The data are the average values of five replicates ± standard error. In the same column, different letters show significant differences between the mean values (*p* < 0.05). CK refers to the untreated mining soil samples, S + M refers to the mining soil samples with the microbial agent, S + SL refers to the mining soil samples with water-jet loom sludge samples mixed at a ratio of 2.5:1, and S + SL × M refers to the mining soil samples with water-jet loom sludge samples and the microbial agent.

**Table 3:** Shannon’s and Chao1 indices of the bacterial and fungal community under the 97% similarity level.

Sample	Chao1		Shannon’s Index	
Bacteria/Fungi	Bacteria	Fungi	Bacteria	Fungi
CK	160.00 ± 4.37 ^a^	102.71 ± 0.14 ^a^	3.84 ± 0.88 ^a^	2.14 ± 0.03 ^a^
S + M	375.63 ± 4.62 ^b^	355.25 ± 2.07 ^c^	6.90 ± 0.56 ^b^	4.47 ± 0.22 ^b^
S + SL	190.32 ± 0.42 ^a^	186.34 ± 1.60 ^b^	3.78 ± 0.66 ^a^	2.19 ± 0.14 ^a^
S + SL × M	715.40 ± 2.30 ^d^	554.84 ± 0.32 ^d^	8.45 ± 0.08 ^c^	5.34 ± 0.12 ^c^

Note: The data are the average values of five replicates ± standard error. In the same column, different letters show significant differences between the mean values (*p* < 0.05).

**Table 4:** Correlation analysis of chemical properties and biological characteristics of the soil.

	SOC	AN	AP	AK	TN	TP	MK	C/N	AGB	MBC	CH
pH	−0.968 *	−0.997 *	−0.991 *	−0.976 *	−0.958 *	−0.871	−0.285	0.966 *	−0.838	−0.971 *	−0.891
SOC		0.953 *	0.980 *	0.989 *	0.999 *	0.980 *	0.367	−0.927	0.954 *	0.994 *	0.988 *
AN			0.991 *	0.984 *	0.951 *	0.876	0.304	−0.949	0.835	0.919	0.902
AP				0.992 *	0.981 *	0.927	0.176	−0.968 *	0.900	0.956 *	0.911
AK					0.985 *	0.940	0.183	−0.929	0.900	0.972 *	0.963 *
TN						0.981 *	0.014	−0.959 *	0.961 *	0.993 *	0.986 *
TP							−0.155	−0.880	0.990 *	0.993 *	0.993 *
MK								−0.092	−0.260	−0.040	−0.059
C/N									−0.883	−0.894	−0.869
AGB										0.969 *	0.997 *
MBC											0.960 *

Note: * *p* < 0.05.

## Data Availability

Not applicable.

## Supplementary Materials

The following are available online at https://www.mdpi.com/article/10.3390/ijerph18136797/s1, Figure S1: USDA classification system of soil texture; Table S1: Properties of soil samples and water-jet loom sludge; Table S2: Experimental method; Table S3: Relative moisture grade of grassland soil in local standards of Inner Mongolia Autonomous Region.

## References

[1] Bian Z., Miao X., Lei S., Chen S.E., Wang W., Struthers S. (2012). The challenges of reusing mining and mineral-processing wastes. Science.

[2] Meng Z.P., Pan J.N., Wang R., Tang C. (2009). Coal Mining Induced Environmental and Geological Problems in China.

[3] Huang J., Wang P., Xu C., Zhu Z. (2018). Fly Ash Modified Coalmine Solid Wastes for Stabilization of Trace Metals in Mining Damaged Land Reclamation: A Case Study in Xuzhou Coalmine Area. Int. J. Environ. Res. Public Health.

[4] Hu Z., Xia Q. (2017). An integrated methodology for monitoring spontaneous combustion of coal waste dumps based on surface temperature detection. Appl. Therm. Eng..

[5] Yu H., Huang J., Ji C., Li Z. (2021). Construction of a Landscape Ecological Network for a Large-Scale Energy and Chemical Industrial Base: A Case Study of Ningdong, China. Land.

[6] Hu Z., Yang G., Xiao W., Li J., Yang Y., Yu Y. (2014). Farmland damage and its impact on the overlapped areas of cropland and coal resources in the eastern plains of China. Resour. Conserv. Recycl..

[7] Bascetin A. (2007). A decision support system using analytical hierarchy process (AHP) for the optimal environmental reclamation of an open-pit mine. Environ. Geol..

[8] Su Y.Z., Zhao W.Z., Su P.X., Zhang Z.H., Wang T., Ram R. (2007). Ecological effects of desertification control and desertified land reclamation in an oasis-desert ecotone in an and region: A case study in Hexi Corridor, northwest China. Ecol. Eng..

[9] Duan L., Liu T., Wang X., Wang G., Ma L., Luo Y. (2011). Spatio-temporal variations in soil moisture and physicochemical properties of a typical semiarid sand-meadow-desert landscape as influenced by land use. Hydrol. Earth Syst. Sci..

[10] Bi Y., Guo Y., Sun H. (2021). Arbuscular mycorrhizal fungal diversity in soils underlying moss biocrusts in coal mining subsidence areas. Environ. Sci. Pollut. Res..

[11] Kolecka K., Gajewska M., Obarska-Pempkowiak H., Rohde D. (2017). Integrated dewatering and stabilization system as an environmentally friendly technology in sewage sludge management in Poland. Ecol. Eng..

[12] Karim A.A., Kumar M., Mohapatra S., Singh S.K. (2019). Nutrient rich rich biomass and effluent sludge wastes co-utilization for production of biochar fertilizer through different thermal treatments. J. Clean. Prod..

[13] Ashekuzzaman S.M., Forrestal P., Richards K., Fenton O. (2019). Dairy industry derived wastewater treatment sludge: Generation, type and characterization of nutrients and metals for agricultural reuse. J. Clean. Prod..

[14] Cehui M., Qitang W., Quanying C., Guirong L., Chengai J. (2000). Utilization of municipal sludge in agriculture and sustainable develppment. Chin. J. Appl. Ecol..

[15] Hanay O., Hasar H., Kocer N.N., Aslan S. (2008). Evaluation for agricultural usage with speciation of heavy metals in a municipal sewage sludge. Bull. Environ. Contam. Toxicol..

[16] Zhang M., Qian Y., Zhou Y., Wu Q., Ma S., Zhou H. (2016). An Optimization Design on Water-Jet Looms Activated Carbon Filter.

[17] Soobhany N. (2019). Insight into the recovery of nutrients from organic solid waste through biochemical conversion processes for fertilizer production: A review. J. Clean. Prod..

[18] Selivanovskaya S.Y., Latypova V.Z., Artamonova L.A. (2003). Use of sewage sludge compost as the restoration agent on the degraded soil of Tatarstan. J. Environ. Sci. Health Part A.

[19] Fijalkowski K., Rosikon K., Grobelak A., Hutchison D., Kacprzak M.J. (2018). Modification of properties of energy crops under Polish condition as an effect of sewage sludge application onto degraded soil. J. Environ. Manag..

[20] Bai Y., Zuo W., Shao H., Mei L., Tang B., Gu C., Wang X., Guan Y. (2018). Eastern China coastal mudflats: Salt-soil amendment with sewage sludge. Land Degrad. Dev..

[21] Ros M., Hernandez M.T., Garcia C. (2003). Bioremediation of soil degraded by sewage sludge: Effects on soil properties and erosion losses. Environ. Manag..

[22] Ciolea D.I., Ionel I., Mihaiuti A. (2019). Research Concerning the Possibility of Turning Sterile Soil Into a Fruitful One, by Using Sludge. Rev. Chim. Bucharest..

[23] Alvarenga P., Rodrigues D., Mourinha C., Palma P., de Varennes A., Cruz N., Tarelho L.A.C., Rodrigues S. (2019). Use of wastes from the pulp and paper industry for the remediation of soils degraded by mining activities: Chemical, biochemical and ecotoxicological effects. Sci. Total Environ..

[24] Fernandes S., Bettiol W., Cerri C.C. (2005). Effect of sewage sludge on microbial biomass, basal respiration, metabolic quotient and soil enzymatic activity. Appl. Soil Ecol..

[25] Zerzghi H., Brooks J.P., Gerba C.P., Pepper I.L. (2010). Influence of long-term land application of Class B biosolids on soil bacterial diversity. J. Appl. Microbiol..

[26] Criquet S., Braud A., Neble S. (2007). Short-term effects of sewage sludge application on phosphatase activities and available P fractions in Mediterranean soils. Soil Biol. Biochem..

[27] Hu Z., Zhu Q., Liu X., Li Y. (2020). Preparation of topsoil alternatives for open-pit coal mines in the Hulunbuir grassland area, China. Appl. Soil Ecol..

[28] Anderson J.D., Ingram L.J., Stahl P.D. (2008). Influence of reclamation management practices on microbial biomass carbon and soil organic carbon accumulation in semiarid mined lands of Wyoming. Appl. Soil Ecol..

[29] Molnar M., Vaszita E., Farkas E., Ujaczki E., Fekete-Kertesz I., Kirchkeszner C., Gruiz K., Uzinger N., Feigl V. (2016). Acidic sandy soil improvement with biochar—A microcosm study. Sci. Total Environ..

[30] Zhang Y., Cao C., Cui Z., Qian W., Liang C., Wang C. (2019). Soil bacterial community restoration along a chronosequence of sand-fixing plantations on moving sand dunes in the Horqin sandy land in northeast China. J. Arid Environ..

[31] Zhang Y., Bi Y., Shen H., Zhang L. (2020). Arbuscular Mycorrhizal Fungi Enhance Sea Buckthorn Growth in Coal Mining Subsidence Areas in Northwest China. J. Microbiol. Biotechn..

[32] NRCS Soils (2010). Keys to Soil Taxonomy.

[33] Shen Z., Xue C., Penton C.R., Thomashow L.S., Zhang N., Wang B., Ruan Y., Li R., Shen Q. (2019). Suppression of banana Panama disease induced by soil microbiome reconstruction through an integrated agricultural strategy. Soil Biol. Biochem..

[34] Dexter A.R. (1988). Advances in characterization of soil structure. Soil Tillage Res..

[35] Yang X.M., Wander M.M. (1998). Temporal changes in dry aggregate size and stability: Tillage and crop effects on as silty loam Mollisol in Illinois. Soil Tillage Res..

[36] Six J., Elliott E.T., Paustian K. (2000). Soil structure and soil organic matter: II. A normalized stability index and the effect of mineralogy. Soil Sci. Soc. Am. J..

[37] McArdle B.H., Anderson M.J. (2001). Fitting multivariate models to community data: A comment on distance-based redundancy analysis. Ecology.

[38] Clauset A., Shalizi C.R., Newman M.E.J. (2009). Power-Law Distributions in Empirical Data. Siam. Rev..

[39] Koroshetz W.J. (1995). Tissue Plasminogen Activator for Acute Ischemic Stroke. N. Engl. J. Med..

[40] Inner Mongolia A.O.Q.S. (2012). Inner Mongolia Farmland, Grassland Relative Humidity Level Indicators.

[41] China M.O.W.R. (2015). Specifications for Soil Moisture Monitoring.

[42] Accoe F., Boeckx P., Busschaert J., Hofman G., Van Cleemput O. (2004). Gross N transformation rates and net N mineralisation rates related to the C and N contents of soil organic matter fractions in grassland soils of different age. Soil Biol. Biochem..

[43] Chen G., Zhu H., Zhang Y. (2003). Soil Microbial Activities and Carbon and Nitrogen Fixation.

[44] R Core Team (2011). R: A language and environment for statistical computing. 2013. Computing.

[45] Qian T., Bagan H., Kinoshita T., Yamagata Y. (2014). Spatial-Temporal Analyses of Surface Coal Mining Dominated Land Degradation in Holingol, Inner Mongolia. IEEE J. Stars.

[46] Ajayi A.E., Horn R. (2016). Comparing the potentials of clay and biochar in improving water retention and mechanical resilience of sandy soil. Int. Agrophys.

[47] Shifeng W.Z.J. (2019). Engineering example of air floatation + bio-contact oxidation process for upgrading treatment of waste water from water jet loom. Environ. Dev..

[48] Ai X., Wang L., Xu D., Rong J., Ai S., Liu S., Li C., Ai Y. (2021). Stability of artificial soil aggregates for cut slope restoration: A case study from the subalpine zone of southwest China. Soil Tillage Res..

[49] Menon M., Mawodza T., Rabbani A., Blaud A., Lair G.J., Babaei M., Kercheva M., Rousseva S., Banwart S. (2020). Pore system characteristics of soil aggregates and their relevance to aggregate stability. Geoderma.

[50] Niu F., Gao Z., Lin Z., Luo J., Fan X. (2019). Vegetation influence on the soil hydrological regime in permafrost regions of the Qinghai-Tibet Plateau, China. Geoderma.

[51] Feng D., Bao W. (2017). Review of the temporal and spatial patterns of soil C: N: P stoichiometry and its driving factors. Chin. J. Appl. Environ. Biol..

[52] Zhang Z.S., Song X.L., Lu X.G., Xue Z.S. (2013). Ecological stoichiometry of carbon, nitrogen, and phosphorus in estuarine wetland soils: In fluences of vegetation coverage, plant communities, geomorpgy, and seawallsu. J. Soils Sediments.

[53] Zhang H., Ouyang Z., Zhao X., Guo X., Ye Y. (2018). Effects of different land use types on soil organic carbon, nitrogen and ratio of carbon to nitrogen in the plow layer of farmland soil in Jiangxi Province. Acta Entiae Circumstantiae.

[54] Gundersen P., Callesen I., de Vries W. (1998). Nitrate leaching in forest ecosystems is related to forest floor C/N ratios. Environ. Pollut..

[55] Stacey N.E., Lewis R.W., Davenport J.R., Sullivan T.S. (2019). Composted biosolids for golf course turfgrass management: Impacts on the soil microbiome and nutrient cycling. Appl. Soil Ecol..

[56] Thompson G.L., Kao-Kniffin J. (2019). Urban Grassland Management Implications for Soil C and N Dynamics: A Microbial Perspective. Front. Ecol. Evol..

[57] Wang L., Chang Y., Liu Q. (2019). Fate and distribution of nutrients and heavy metals during hydrothermal carbonization of sewage sludge with implication to land application. J. Clean. Prod..

[58] Kitz F., Gomez-Brandon M., Eder B., Etemadi M., Spielmann F.M., Hammerle A., Insam H., Wohlfahrt G. (2019). Soil carbonyl sulfide exchange in relation to microbial community composition: Insights from a managed grassland soil amendment experiment. Soil Biol. Biochem..

