# Supplementary material for: Feasibility Study on the Application of Microbial Agent Modified Water-Jet Loom Sludge for the Restoration of Degraded Soil in Mining Areas
Source: Int J Environ Res Public Health. 2021 Jun 24;18(13):6797. doi: 10.3390/ijerph18136797 (PMC8296874; doi:10.3390/ijerph18136797)
Supplement: Supplementary file 1 [file ijerph-18-06797-s001.zip › ijerph-1179955-supplementary.pdf]

Table S1 Properties of soil samples and water-jet loom sludge

Test materials	pH	TN (mg/kg)	TP (mg/kg)	MK (mg/kg)	SOC (g/kg)	AN (mg/kg)	AP (mg/kg)	AK (mg/kg)	MBC (mg/kg)
Soil	8.96±1.25	558±31.4	344±20.2	2.18±0.32	0.19±0.05	8.72±0.24	1.50±0.00	33.0±1.80	25.0±5.0
Sludge	6.49±0.88	6800±108	3280±258	8.10±1.56	11.59±0.86	152.7±10.58	33.90±3.60	89.6±13.9	1234±189
Heavy metal content (mg/kg)	Pb	Cr	Ni	Cu	Zn	Cd			
Soil	38.57±9.42	88.54±8.02	40.96±2.79	51.52±2.89	123.1±9.56	0.616±013			
Sludge	ND	ND	0.05±0.02	ND	0.385±0.54	ND			

Note: The values were presented as mean ± standard error of mean (S.E.M).; TN: total nitrogen; TP: total phosphorus; MK: mineral potassium; SOC: total C content; AN: available nitrogen; AP: available phosphorus; AK: available potassium; MBC: microbial biomass C; ND means no detection.

Table S2 Experimental method

**Target**	**Determination method**	**Main reagents**	**Basis**
MK	Flame photometry	HF-$HClO_4$, Na_2O standard solution	LY/T1254-1999
TP	Alkali fusion-Mo-Sb anti spectrophotometry	H_2SO_4, NaOH, CH_3CH_2OH, HNO_3	HJ632-2011
TN	Kjeldahl method	H_2SO_4, H_3BO_3, HCl standard solution	HJ717-2014
SOC	Combustion oxidation nondispersive infrared absorption	H_3PO_4, CO_2-free water	HJ695-2014
AN	NaOH-hydrolyzation diffusion	NaOH, H_3BO_3	\
AP	$NaHCO_3$ extraction	0.5 M $NaHCO_3$, acid molybdenum-antimony in color mixed	\
AK	NH_4OAc extraction	NH_4OAc, KCl, 1N CH_3COONH_4	\
MBC	Fumigation-extraction	K_2SO_4, $K_2Cr_2O_7$	\
Cr, Pb, Ni, Cu, Zn, Cd	Inductively coupled plasma mass spectrometry	HCl, HNO_3 (2 + 98, 5 + 95), 30% H_2O_2, HF	HJ766-2015
Se	Atomic fluorescence spectrometry	HNO_3, $HClO_4$, HCl	NY/T 1104-2006
Hg, As	Atomic fluorescence spectrometry	HCl, CH_4N_2S solution, As standard working solution	GB/T22105.2-2008

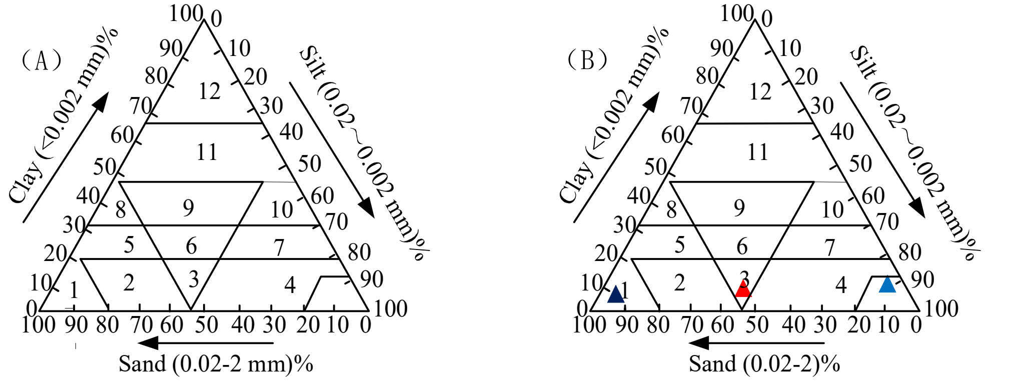

1. Sand and loamy sand 2. Sandy loam 3. Medium loam 4. Silt loam 5. Sandy clay loam 6. Clay loam 7. Silty clay loam 8. Sandy clay 9. Loamy clay 10. Silty clay 11. Clay 12. Heavy clay

▲ Mining area soil ▲ Textile sludge

▲ Mixture of textile sludge and mining area soil at a mass ratio of 1:2.5

Fig S1 USDA classification system of soil texture [32] (A). Location of mining area soil, water-jet loom sludge, and a mixture of water-jet loom sludge and mining area soil on the USDA classification system of soil texture (B).

Table S3 Relative moisture grade of grassland soil in local standards of Inner Mongolia Autonomous Region [34]. According to the relative humidity of soil to determine the type of soil in grassland, also described the basic properties of this kind of soil.

Soil moisture types of grassland	Relative humidity of soil（%）	soil properties
Excessive moisture content	>90	The relative humidity of soil is close to or higher than the capacity of grassland, and it is close to saturation and is muddy
First class of soil moisture	70~90	The soil relative humidity is lower than the field water holding capacity and higher than the maximum molecular water holding capacity
Second class of soil moisture	50~70	The relative humidity of soil is close to the maximum molecular water holding capacity, and it has the feeling of semi humid
Third class of soil moisture	30~50	The relative humidity of the soil is close to or higher than the withering point, and the soil is moist or slightly humid
Extremely dry soil moisture	<30	The relative humidity of the soil is close to or lower than the withering point, and there is no sense of moisture. When the wind blows, it can cause dust
